# Growth dynamics of plexiform neurofibromas: a retrospective cohort study of 201 patients with neurofibromatosis 1

**DOI:** 10.1186/1750-1172-7-75

**Published:** 2012-10-04

**Authors:** Rosa Nguyen, Eva Dombi, Brigitte C Widemann, Jeffrey Solomon, Carsten Fuensterer, Lan Kluwe, Jan M Friedman, Victor-Felix Mautner

**Affiliations:** 1 Department of Neurology, University Hospital Hamburg-Eppendorf, Hamburg, Germany; 2 Department of Pediatrics, University of Maryland, 22 South Greene Street, Baltimore, MD, 21201, USA; 3 Pediatric Oncology Branch, National Cancer Institute, Bethesda, USA; 4 Department of Radiology, MRI Institute Hamburg Othmarschen, Hamburg, Germany; 5 Department of Maxillo-Facial-Surgery, University Hospital Hamburg-Eppendorf, Hamburg, Germany; 6 Department of Medical Genetics, University of British Columbia, Vancouver, Canada

**Keywords:** MRI scan, Neurofibroma, Plexiform, Neurofibromatosis 1, Tumour burden

## Abstract

**Background:**

To examine the natural growth dynamics of internal plexiform neurofibromas (PNs) in patients with neurofibromatosis 1 (NF1).

**Methods:**

Two hundred and one NF1 patients underwent whole body MRI (WBMRI). Tumour burden was estimated volumetrically. Non-parametric Spearman’s rho correlation coefficients were used to analyse the relationship of growth rate to tumour volume and age. Chi-squared and Mann–Whitney U tests were used for analysing the association of tumour occurrence with sex or age. Chi-squared tests were used to analyse the association of tumour growth with age group.

**Results:**

Seventy-one of 171 patients with serial WBMRI exams had internal PNs (median follow up 2.2 years [1.1 to 4.9 years]). Median whole body tumour volume was 86.4 mL [5.2 to 5878.5 mL]) with a median growth rate of 3.7%/year (−13.4 to 111%/year) that correlated with larger whole body tumour volume (*P*<0.001) and lower age (*P*=0.004). No new PNs developed in 273.0 patient-years among patients without tumours. Rate of new tumour development among patients with PNs was 0.6%/year (95% confidence interval 0.02 to 3.4%). Twenty-seven (13.5%) tumours increased significantly and were more frequent among children (*P*<0.001). Growth rate of tumours was inversely correlated with age (Spearman’s rho=−0.330, *P*<0.001). Seventy-one (35.5%) tumours had smaller volumes on follow up (median −3.4%/year [−0.07% to −35.9%/year]).

**Conclusion:**

Children with NF1 and internal PNs are at risk for tumour growth. Most PNs grow slowly or not at all, and some decrease in size. New tumours are infrequent in NF1 patients with PNs and unlikely in patients without PNs.

## Background

Plexiform neurofibromas (PNs) develop frequently in individuals with neurofibromatosis 1 (NF1). Physical examination can detect superficial PNs in about 27% of NF1 patients but internal PNs can only be detected by imaging studies such as magnetic resonance imaging (MRI) [1,2]. Our cross-sectional study with whole body MRI demonstrated internal PNs in 50% of NF1 patients [3]. Recent studies revealed that 55% of PNs in childhood are symptomatic [4]. About 17% of superficial PNs, 38% of displacing PNs, and 64% of invasive PNs cause functional impairment [5]. PNs may also transform into malignant peripheral nerve sheath tumours (MPNSTs), which occur in about 10% of NF1 patients [6]. Higher internal tumour burden appears to be a risk factor for the development of MPNST, and rapid increase in size, the development of severe pain, or new neurological deficits may indicate malignant transformation of an existing PN [3,6].

Growth of PNs varies greatly among patients but its course over time has not been well delineated [7,8]. Characterizing the natural history of PN growth is essential for patient management as well as for designing clinical trials. Better understanding of the growth dynamics of PNs may also help identify patterns that are indicative of malignant transformation.

Results of two recent studies suggest that the growth of PNs is inversely correlated with age, at least in younger NF1 patients [7,8]. However, one of these two studies assessed PN growth using two-dimensional measurements [7], that are relatively insensitive to changes in tumour size [9]. The other study used three-dimensional volumetric analyses but included only selected tumours in a small number of children and adolescents, who were enrolled for drug trials [8]. In the present study, we assessed internal PNs by volumetric whole body MRI in 171 unselected NF1 patients of various ages and followed tumour growth over a median period of 2.2 years.

## Methods

### Patients

This is a retrospective cohort study. A total of 201 NF1 patients diagnosed according to the National Institutes of Health Diagnostic Criteria National Institutes of Health [10], who underwent whole body MRI between 2003 and 2008 were enrolled in this study. We did not exclude patients, who had prior surgery, but MRI examinations on participants, who underwent surgery during the period of study were excluded. The Ethical Committee of the Medical Chamber in Hamburg approved the investigation. All patients gave informed consent to participate.

Whole body axial short tau inversion recovery (STIR) MRI studies were acquired using a 1.5 Tesla Siemens Magnetom 63 SP/Symphony/Avanto scanner. Images were obtained from the top of the head to mid-calf in a series of ten mm slices without skips.

PNs were diagnosed on the basis of their characteristic appearance as signal intense masses on T2-weighted STIR MRI sequences [11]. PNs smaller than 3.0 cm in greatest diameter were excluded because their volume could not be assessed accurately on the whole body MRI images.

### Volumetric MRI analysis

Analysis of tumour volume was performed using MedX software (v3.42), a heuristics-based semi-automated method for segmentation and measurement [9]. Difference of signal intensity in healthy (low intensity) and tumour (high intensity) tissue was utilized to define tumour margins on each axial slice and thereafter to perform an automated volume calculation. This volumetric assessment is sensitive and reproducible, yielding results similar to those of manual tracings [9]. If automated border tracing was not possible for a particular tumour, manual tracing was performed.

### Statistical analyses

Because of the irregular distribution of age, tumour number, tumour size, and tumour growth rate, non-parametric Spearman’s rho correlation coefficients were used to analyse the relationship of growth rate to tumour volume and age. Chi-squared and Mann–Whitney U tests were used for analysing the association of tumour occurrence (presence or absence on whole body MRI) with sex or age, respectively. We defined an increase in tumour volume that exceeded 20% per year compared to the previous volume as significant tumour growth in accordance with previous studies using this method [9]. Children were defined as individuals below 18 years of age, and adults as those 18 of age and older. Chi-squared tests were used to analyse the association of tumour growth to age group. All calculations were performed with SPSS Predictive Analytics Software version 18.0 (IBM Corporation, Armonk, New York, USA). p ≤ 0.05 was considered to be statistically significant.

## Results

### Analysis of whole body tumour burden

Two hundred one NF1 patients (113 females and 88 males) were enrolled in this study. The median age at the time of initial MRI was 28.6 years (range 1.7-63.4 years). A total of 100 (49.8%) of the 201 patients studied at the initial whole-body MRI had one or more internal PN, while the other 101 patients (50.2%) did not have any measurable internal tumours. There was no significant association between the presence of tumours and age, but a weak correlation was seen with male sex (Spearman’s rho=0.145, *P*=0.041).

Thirty patients, who received a baseline MRI could not be included in the whole body longitudinal analysis because they did not return for follow-up examination, they had surgery on one or more tumours after their initial MRI, or imaging quality on follow-up MRI was not adequate for volumetric analysis of all tumours measured on the first examination. Of the 171 patients, who had serial whole body MRI scans analysed, 140 patients (81.9%) had two MRI examinations, 29 patients (17.0%) had three scans, and two patients (1.2%) had four scans. Altogether, a total of 375 whole body MRI scans on these 171 patients were analyzed. The median duration of follow up was 2.2 years, ranging from 1.1 to 4.9 years.

**Figure 1:**
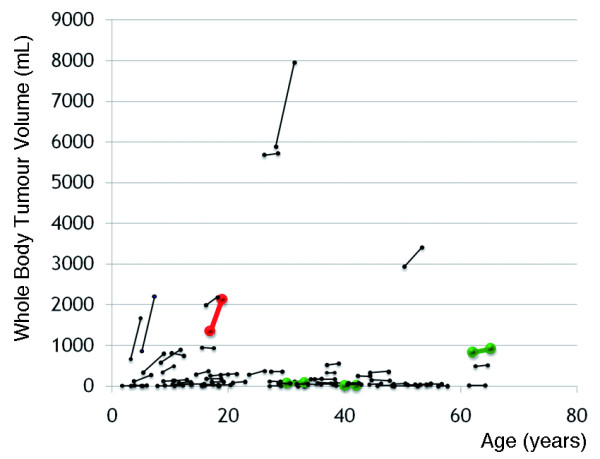
**Whole body tumour volume by person and age; whole body tumour volume by person and age in 71 patients with NF1.** Patient 1890, who developed MPNST during the course of the study, is shown in red. Patients 1140, 1730, and 2490, who were treated for MPNSTs prior to their entry into the study, are shown in green.

Seventy-one of the 171 NF1 patients, who had serial whole body MRI examinations had internal PNs. In these 71 patients, the median total body volume of tumours was 86.4 mL (range, 5.2-5878.5 mL) on initial exam. The median rate of growth in whole body tumour burden expressed as a percentage of the total volume of tumours measured in the patient on first exam was 3.7% per year, with a range of −13.4% to 111.1% per year. Figure 1 shows the change in whole body tumour volume by age in each of the patients with internal PNs, who underwent serial whole body MRI (cf. Additional file 1).

In these 71 patients, the whole body tumour volume at the time of the initial MRI scan correlated with the absolute rate of growth of the whole body tumour burden (expressed as mL/year) (Spearman’s rho = 0.499, *P*<0.001). Age at first exam correlated inversely with whole body tumour growth rate expressed as a percentage of the initial total tumour volume (Spearman’s rho = −0.340, *P*=0.004).

**Figure 2:**
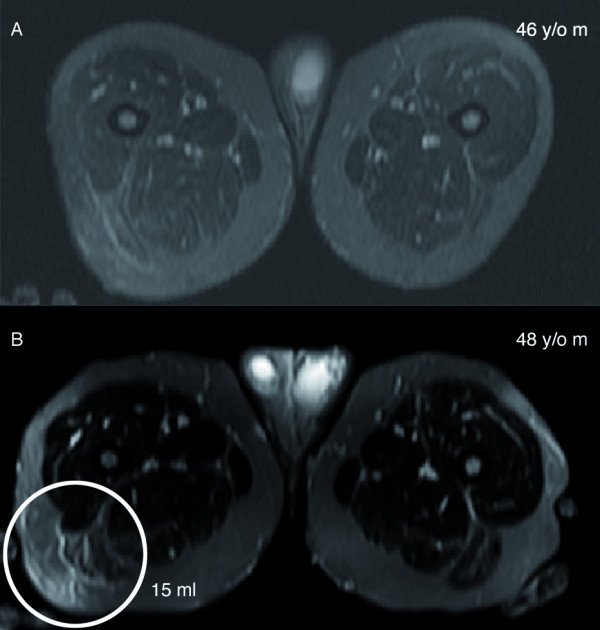
**Development of a new PN on MRI; the only PN that arose during the period of observation in this study is shown in this figure (Patient 1670, Tumour 5). A**: cross-sectional image through the thigh at age 46 years; **B**: cross-sectional image through the thigh at age 48 years.

No new PNs (95% Poisson exact confidence interval, 0.0 to 3.69 per year) developed in 273.0 patient-years of observation among the 100 patients, who had no detecf tumours on initial MRI. The risk of new internal PN development among these patients is likely to be <1.4% per year. Among the 71 patients with tumours on initial MRI, who were followed with serial examinations, one (95% Poisson exact confidence interval, 0.03-5.57) new tumour arose in 165.5 patient-years of observation (Figure 2). The risk of new tumour development among patients with one or more PNs on initial whole body MRI was 0.6% (95% confidence interval, 0.02-3.4%) per year.

### Analysis of growth of individual tumours

Volumetric MRI measurements on at least two occasions were available on a total of 201 different tumours in 95 NF1 patients, who had one or more tumours identified on the first examination. Sixty-six tumours were identified among 32 patients, who were younger than 18 years of age at first exam. A total of 135 PNs were found in 63 adults (age 18 years or older).

**Figure 3:**
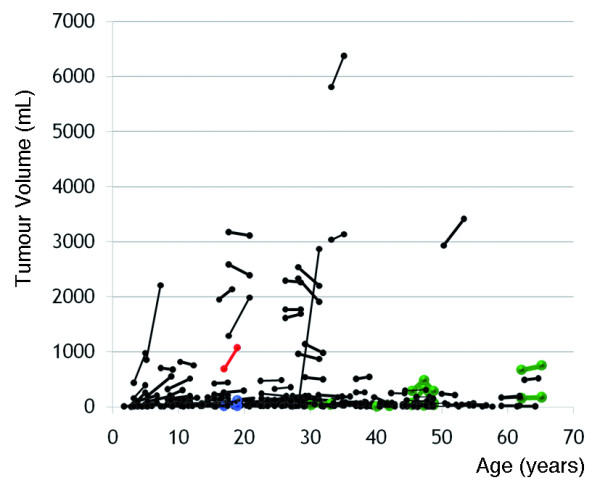
**Tumour volume of individual tumours by age; tumour volume of 201 individual tumours by age in 95 patients with NF1.** The MPNST that developed in Patient 1890 during the course of the study is shown in red. The other two tumours that were seen in Patient 1890 are shown in blue, and six tumours that were followed in Patients 1140, 1730 and 2490, who were treated for MPNSTs prior to their entry into the study, are shown in green.

The median volume of these 201 tumours on the first valid MRI exam was 44.3 mL, with a range of 0–5810.4 mL. One tumour was not seen on the subject’s first MRI but was apparent and measurable on the follow-up exam (Patient 1670, tumour 5); this tumour was excluded from the analyses of tumour growth. Figure 3 shows the change in volume of the 201 individual tumours by age in 95 NF1 patients, who underwent serial whole body MRI (cf. Additional file 2).

The median rate of growth of the individual tumours expressed as a percentage of the volume measured on the first exam was 2.8% per year, with a range of −35.9% to 3667% per year. The rate of growth of individual tumours was inversely correlated with age at initial examination (Spearman’s rho = −0.330, *P*<0.001) but not with the volume of tumour on initial MRI exam.

Twenty-seven (13.5%) of the 200 individual tumours that were followed longitudinally by MRI in this study increased in size more than 20% per year, on average. Among these 27 PNs, 19 were present in patients younger than 18 years of age, corresponding to 29% of the 66 tumours found in this group of patients. Eight (6.0%) of 134 tumours among patients older than 18 years grew more than 20% per year. Tumours that grew more than 20% per year were significantly more frequent among children than among adults (*P*<0.001).

One of the 19 PNs in children under 18 that increased in size more than 20% per year during the period of observation underwent transformation into a MPNST (Patient 1890, tumour 3). One of the other two tumours in this patient (Patient 1890, tumour 1) grew more than 20% per year during the period of observation, but none of six benign tumours studied in four patients (1140, 1180, 1730, and 2490), who had had MPNSTs treated prior to entry into this study grew more than 20% per year. All of these patients were over 30 years of age at the time of entry into the study.

Seventy-one (35.5%) of the 200 internal PNs followed longitudinally by MRI had smaller volumes measured on follow up than on initial exam. Among these 71 tumours, the median measured change in volume was −3.4% per year, and the range was −0.07% to −35.9% per year in comparison to the volume measured on the first MRI exam. One hundred twenty-six (63.0%) of the tumours had larger volumes measured on follow-up MRI than on the initial scan. The median increase in volume per year among these 126 tumours was 7.4%. The range was 0.24% to 3667% per year.

## Discussion

This is the first reported longitudinal study with whole body MRI and three-dimensional volume measurement of internal PNs in an unselected group of NF1 patients. We see about 1200 individuals with NF1 each year at the NF Centre Hamburg, and patients are referred to us by physicians throughout Germany and through the German patient lay organization. We see a broad phenotypic spectrum; however, because of our recognized expertise, it is likely that more severe and complex cases are preferentially referred to our centre. Patients were asked consecutively to participate in the study between 2003 and 2008 regardless of gender, age, or clinical presentation, but only a fraction of these patients agreed to undergo WBMRI exam and gave written informed consent. The main reasons for patients or guardians to refuse participation were logistical problems, claustrophobia, the need for moderate sedation in children, and expected discomfort. It is possible that patients with a more severe course or, who were more concerned about the possibility of internal tumours were more likely to volunteer for the study.

Our results clearly show that the growth rate of PNs in NF1 patients is correlated with whole body total tumour volume and inversely correlated with age. The greater rate of growth in young children is consistent with clinical observations and with the results of two previous studies in NF1 patients [7,8]. Individuals with high whole body tumour volumes and young patients deserve close monitoring by clinical examination and whole body MRI. Because their tumours are more likely to grow, early surgical intervention may be considered [12]. Older patients and those with no internal tumours on initial MRI are less likely to develop neurological or other complications that are associated with large internal tumours or tumour growth. Further investigations are needed to see if more frequent monitoring of high-risk patients and less frequent monitoring of low-risk patients would improve care and over-all survival in NF1 patients.

Some of the patients included in our study had undergone surgery for removal or reduction of tumours in the past. Surgical reduction of tumours may have an influence on their growth [13]; this needs to be explored in future studies.

PNs that transform into MPNSTs often have growth rates that exceed those of most PNs. The only MPNST that arose during observation in this cohort (Patient 1890, tumour 3) showed a growth rate of 28% per year. The malignancy was diagnosed at 18.9 years of age after two years of follow up. The only NF1 patient, who developed an MPNST in our previous longitudinal study of internal tumours using local 2-dimensional MRI measurements was also the only adult in that study whose tumour exhibited rapid growth [7]. Since PNs usually grow slowly, if at all, in adulthood, adults with progressive tumours on serial MRI exams raise high suspicion for an MPNST and should undergo further diagnostic assessment by positron emission tomography or biopsy [14].

Benign plexiform neurofibromas may grow rapidly in children and adolescents (Figure 3). Children and young adults with rapidly growing tumours or large total body tumour burden may be at especially high risk for developing an MPNST in the future, but longitudinal whole body MRI studies with longer follow up are needed to determine whether this is so. We note with some concern that whole body tumour burdens greater than 1000 mL are distinctly unusual in the patients we studied over 40 years of age.

Patients with no internal tumours on first MRI examination are unlikely to develop new PNs later. This is consistent with the observation that clinically apparent PNs rarely develop in NF1 patients after early childhood [15,16]. However, new PNs may develop, or at least first become apparent, later in life in people with NF1, who do have internal tumours, although this is not frequent. One previous longitudinal MRI study of NF1 patients reported the incidental detection of a new PN but could not draw any conclusions regarding the likelihood of new tumour formation [7]. MRI cannot determine when the molecular process of tumourigenesis began, and the rate of growth of tumours that are large enough to image does not necessarily have any relationship to their rate of growth earlier in tumour development. Therefore, we do not know if the molecular process that leads to formation of PNs always occurs before birth or if it can also take place later in life.

An important implication of our findings for therapeutic research in NF1 is that it may be best to design clinical trials for treatment of PNs to target pediatric patients, in whom either reduction of growth rate or regression of the tumour can be taken as outcome measures. For trials targeting adult patients, only tumour regression can be taken as an outcome measure because substantial growth of PNs is uncommon.

We found that 35.5% of the tumours in this study showed apparent decreases in volume during the period of observation without any medical or surgical intervention. The median decrease in the volume measurements for these tumours was 3.4% per year, with a range of 0.07% to 35.9% per year. It seems likely that many of these tumours did not actually change in size over the period of observation, and the difference observed represents measurement error [9]. In other instances, tumour shrinkage may have occurred as observed with clinical measurements during our practise. Further studies are needed to determine the magnitude of measurement variability in volumetric measurements of PNs obtained by MRI and the frequency and clinical implications of true tumour shrinkage, if it occurs. In any case, the possibility of apparent decrease in tumour size over time needs to be considered when evaluating therapeutic efficacy and treatment success in clinical trials.

## Conclusions

In summary, whole body MRI is a valuable tool in assessing internal tumour volume and growth of PNs in people with NF1. Patients with high total tumour volume and young age were found to have tumour growth that might lead to complications in the future. On the other hand, patients without internal tumours on their first whole body MRI appear unlikely to develop PNs and associated complications later on. Long-term natural history studies with serial longitudinal whole body MRI examinations and clinical evaluation are needed to provide the evidence necessary for improving the care of people with NF1.

## Abbreviations

MPNST: Malignant peripheral nerve sheath tumor; MRI: Magnetic resonance imaging; NF1: Neurofibromatosis 1; PN: Plexiform neurofibroma; STIR: Short tau inversion recovery; WBMRI: Whole body magnetic resonance imaging.

## Competing interests

The authors report no competing interests.

## Authors’ contribution

RN conducted the volumetric analysis and drafted the manuscript. ED and BCW designed the study and revised the volumetric data. JS designed the volumetric software and gave technical support. CF reviewed the MRI scans radiologically. LK helped to draft the manuscript. JMF performed the statistical analysis and revised the manuscript. VFM revised the manuscript and is the guarantor for the study. All authors read and approved the final manuscript.

## Supplementary Material

Additional file 1 **Tabular data.** Whole body tumour burden by person. This table summarizes clinical data of whole body tumour burden by person for each time point of observation. (XLS 50 kb) Click here for file

Additional file 2 **Tabular data.** Single tumour by person. This table summarizes clinical data for every single tumour by person for each time point of observation. Click here for file
